# Investigation into Improving the Water Resistance and Mechanical Properties of Calcined Gypsum from Phosphogypsum Composites

**DOI:** 10.3390/ma18122703

**Published:** 2025-06-09

**Authors:** Qing Wang, Yuanyuan Lou, Yanzhou Peng, Weiqi Wang, Xiaohui Luo, Abutu Simon John Smith

**Affiliations:** 1 Hubei Engineering Technology Research Center for Disaster Prevention and Mitigation, China Three Gorges University, Yichang 443002, China; postwq@163.com; 2 College of Civil Engineering and Architecture, China Three Gorges University, Yichang 443002, China; louyyuan@163.com (Y.L.); postlxh_9@163.com (X.L.); 3 Wuhan Zhiyuan Construction Group Co., Ltd., Wuhan 430000, China; lmy123456qwe@163.com; 4 Department of Civil Engineering, Federal University of Technology, Babura 732104, Nigeria; abutusmith@gmail.com

**Keywords:** calcined gypsum from phosphogypsum, water resistance, strength, water contact angle, nano-SiO_2_, kaolin, sodium methyl silicate

## Abstract

This study aimed to improve the mechanical properties and water resistance of calcined gypsum from phosphogypsum (CGP) by incorporating organic additives and inorganic admixtures. The effects of the dosage of these additives—including kaolin, nano-SiO_2_, polycarboxylic acid superplasticizer, and sodium methyl silicate—on the properties (flexural strength, compressive strength, water absorption, and softening coefficient) of CGP composites (CGPCs) were investigated. A high water resistance of the CGPCs was achieved using nano-SiO_2_ and sodium methyl silicate modification, superplasticizer addition, and the partial replacement of gypsum with mineral admixtures. The results showed that the flexural and compressive strength of the composites hit 4.61 MPa and 19.54 MPa, respectively, while the softening coefficient was 0.70 and the water absorption rate was 19.85%. Microstructural investigation confirmed that the combination of nano-SiO_2_ and kaolin led to the formation of calcium silicate hydrate. Additionally, the superplasticizer played a crucial role in reducing the water-to-cement ratio, while unhydrated mineral particles had a filling effect, thereby enhancing the density of the hardened paste. The sodium methyl silicate formed a hydrophobic film on the surface of the hardened paste, increasing the contact angle to 109.01° and improving the water resistance of the CGPCs.

## 1. Introduction

Phosphogypsum (PG) is an industrial byproduct of wet phosphoric acid production, and approximately four to five tons of PG are generated for every ton of phosphoric acid production [1,2]. Like natural gypsum, phosphogypsum mainly comprises calcium sulfate dihydrate (CaSO_4_·2H_2_O). Nevertheless, it also contains impurities of residual free phosphoric acid, phosphates (CaHPO_4_·2H_2_O, CaHPO_4_·H_2_O, and Ca_3_(PO_4_)_2_), fluorides, trace metals, and organic matter that adhere to the surface of gypsum crystals and are substituted in the crystal lattice of gypsum [3,4,5,6,7]. The impurities directly affect the performance of phosphogypsum, significantly hindering extensive PG recycling and utilization. The majority of the discharged PG is still disposed of in large stockpiles, resulting in serious environmental contamination of soil, water, and the atmosphere [5,8,9,10,11,12]. At present, approximately 80 million tons of PG are produced annually in China, and it is estimated that the total stock of phosphogypsum in China exceeds 300 million tons [5]. Therefore, the recycling and comprehensive utilization of this byproduct has economic and environmental benefits.

A great deal of research has been conducted on using PG in different fields such as soil stabilization amendments, agricultural fertilizers, retarders in Portland cement, and building materials [5,9,13,14,15,16,17,18,19]. Ancient and extensively used in dry walls, gypsum plaster may be the most promising material for recycling and reusing phosphogypsum. Gypsum plaster can be made by calcinating natural gypsum or its industrial by-products, such as phosphogypsum and flue gas desulfurization gypsum. However, compared to calcined gypsum from natural gypsum, calcined gypsum from phosphogypsum is restricted due to its lower performance, including weak mechanical properties and poor water resistance, which mainly results from the impurities in phosphogypsum [5,9].

This study aimed to improve the water resistance and mechanical properties of calcined gypsum from phosphogypsum. In general, the poor water resistance of calcined gypsum (or gypsum plaster) is thought to be attributed to the following three factors [16]. First, gypsum products possess a high porosity, resulting from the excess water added during the preparation process. Second, calcium sulfate dihydrate, the hydration product of calcined gypsum, is characterized as a hydrophilic substance. Consequently, gypsum products exhibit a propensity to absorb moisture in humid environments. Third, calcium sulfate dihydrate exhibits solubility. For instance, at 20 °C, its solubility in water is measured at 2.04 g/L [20,21]. This implies that gypsum crystals, particularly those situated at the contact points within hardened paste, are prone to dissolution when exposed to moisture or water. Such dissolution can compromise the intercrystalline bonding and consequently lead to a reduction in the strength of the products.

**Figure 1:**
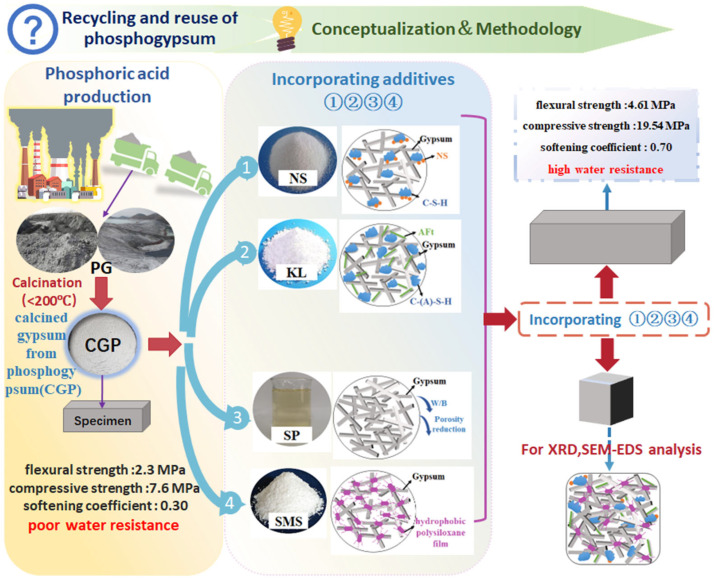
Conceptualization and methodology of the present study.

Therefore, enhancing the water resistance of gypsum plaster could be achieved by the addition of a water reducer, modification with a waterproof agent, and the partial replacement of calcined gypsum with mineral admixtures. The literature has demonstrated that mineral admixtures, such as siliceous materials (e.g., silica fume), aluminosilicate materials (including metakaolin, ground granulated blast-furnace slag, and Portland cement), can be employed to improve both the water resistance and the strength of natural gypsum, fluorgypsum, and flue gas desulfurization gypsum [21,22]. The present study investigated achieving high water resistance in calcined phosphogypsum composites, thereby facilitating the recycling and reuse of phosphogypsum. The literature indicates that mineral admixtures, such as silica fume and granulated blast-furnace slag, are less effective than kaolin in enhancing the properties of calcined gypsum from phosphogypsum composites (CGPCs) [23,24,25,26,27]. Similarly, additives such as polymethyltriethoxysilane are less effective than sodium methyl silicate in improving the water resistance of CGPCs [23,28]. Based on these findings, and after comprehensive consideration of factors including mechanical performance, water resistance, and economic cost, kaolin and sodium methyl silicate were ultimately selected as the additives to improve the performance of CGPCs. This research focused on evaluating the water resistance and mechanical properties (flexural and compressive strength) of these composites following the incorporation of mineral admixtures, specifically nano-SiO_2_ and kaolin combined with hydrated lime, as well as polycarboxylate superplasticizer and sodium methyl silicate. Initially, the effects of the contents of nano-SiO_2_, kaolin, polycarboxylate superplasticizer, and sodium methyl silicate on the properties of calcined phosphogypsum composites were studied. Subsequently, the enhancement mechanisms by which these additives improve the strength of calcined phosphogypsum were explored using X-ray diffraction (XRD), scanning electron microscopy (SEM), and water contact angle tests. The conceptualization and methodology of the present study are shown in Figure 1.

## 2. Experimental Programs

### 2.1. Materials

**Figure 2:**
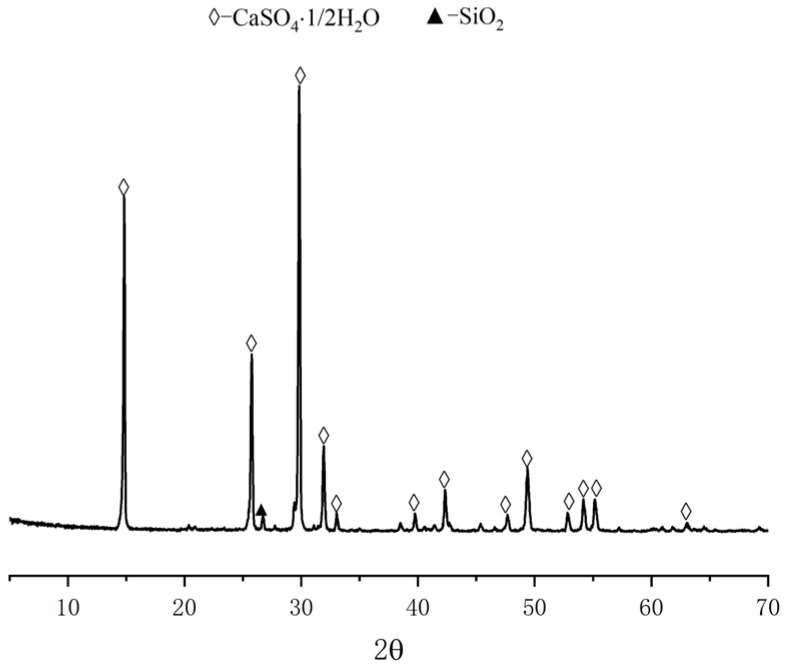
XRD pattern of CGP.

**Figure 3:**
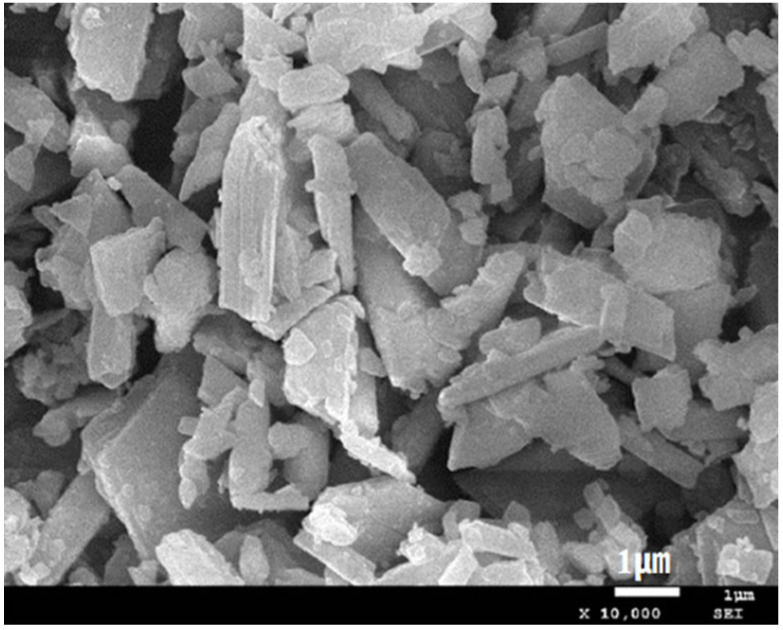
SEM image of CGP (×10,000).

The calcined phosphogypsum composites considered here were prepared from the following ingredients. Calcined gypsum from phosphogypsum (CGP) was obtained by calcination of phosphogypsum (PG) from Hubei Yihua Group, Yichang, China, after being pretreated with lime neutralization in an oven at 150 °C for 6 h. The CGP XRD and SEM analysis results are shown in Figure 2 and Figure 3. Calcium hydroxide (Ca(OH)_2_) was produced by Tianjin Zhiyuan Chemical Reagent Co., Ltd., Tianjin, China, with a Ca(OH)_2_ content of 95.8%. Nano-SiO_2_ (NS) was a white powder with an average particle size of 20 nm, a specific surface area of 240 m^2^/g, and a density of 2.4 g/cm^3^. Kaolin (KL) was a white powder, produced by Henan Yixiang New Materials Co., Ltd., Zhoukou, China. The chemical compositions of CGP and KL are shown in Table 1. The superplasticizer (SP) was provided by Shanxi Qinfen Building Materials Co., Ltd., Yuncheng, China. Its solid content was 40%, and the water reduction rate was 35%. Sodium methyl silicate (SMS) was produced by Shandong Yousuo Chemical Technology Co., Ltd., Heze, China. Its solid content was 30%, and its specific gravity was 1.2.

### 2.2. Sample Preparation and Test Methods

The present study examined the effects of additives (mineral and chemical), including NS, KL, SP, and SMS, on the properties of calcined gypsum from phosphogypsum composites (CGPCs). The mix ratios of the CGPCs are provided in Table 2. As shown in the table, KL-5 denotes a 5% dosage of kaolin (by the total mass of cementitious ingredients), while NS-0.5 refers to a 0.5% content of NS (by the total mass of cementitious ingredients). When KL was added to the CGPCs, the same dosage of CH was simultaneously incorporated.

For each composite, all components (CGP, KL, CH, additives, and water) were mixed, cast, and vibrated in a sequence similar to that of conventional gypsum plaster, and specimens (40 mm × 40 mm × 160 mm) were then prepared and cured following the Chinese Standard GB/T 17669.3-1999 “Gypsum plasters—Determination of mechanical properties” [29].

The flexural and compressive strengths of the specimens were measured according to GB/T 17669.3-1999 [29]. Specifically, the flexural strength was calculated as the average of the measurements obtained from three prism specimens (40 mm × 40 mm × 160 mm), while the compressive strength was determined as the arithmetic mean of the results from three specimens. To determine the water absorption, the specimens were dried to constant weight in a blast electric oven at 40 °C, and then immersed in water at 20 ± 2 °C for 24 h. The weight change of the specimens before and after immersion was then calculated to characterize the water absorption, which was also derived as the arithmetic mean of the values obtained from three prism specimens. As for the softening coefficient, the compressive strength of the specimens after measuring water absorption was tested, before calculating the softening coefficient by dividing the value by the compressive strength of the dry specimens before water absorption.

Some CGPCs were selected for microscopic investigation (Section 3.5). The samples were splinters taken from the prism specimens that had been used for compressive strength testing. The splinter samples were immersed in ethanol for at least 24 h to dehydrate and were then oven-dried at 40 °C for 24 h. Finally, some splinter samples were ground to a fine powder of approximately 10 μm for XRD analysis, and the rest of the splinter samples were utilized to perform SEM analysis after vacuum metal spraying.

XRD spectra were carried out using an AXS D8-Advance X-ray diffractometer (Bruker, Karlsruhe, Germany). A scan range of 3°–70° (2 theta) was selected, and the scan step was 0.02°. SEM analysis was conducted using an ULTRA PLUS scanning electron microscope (JSM-7500F, JEOL, Tokyo, Japan) at an accelerating voltage of 15 kV. Additionally, the water contact angle of the oven-dried specimens was measured using an automatic contact angle tester (Theta Lite, Biolin Scientific, Espoo, Finland). Two parallel samples were set for each CGPC, and the mean value of the two parallel samples was taken as the final contact angle.

## 3. Results and Discussion

### 3.1. Effect of Inorganic Admixtures (Kaolin and Nano-SiO_2_) on the Properties of the CGPCs

#### 3.1.1. Effect of the Nano-SiO_2_ Content

**Figure 4:**
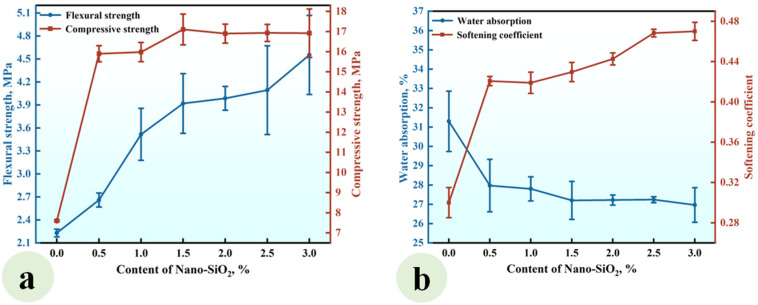
Effect of the content of nano-SiO_2_ on (**a**) the strength and (**b**) water absorption and softening coefficient of the CGPCs.

A series of CGPCs (Series NS) with different nano-SiO_2_ contents (0, 0.5%, 1.0%, 1.5%, 2.0%, 2.5%, and 3.0%) were prepared using the formulations shown in Table 2. The calcium hydroxide content was fixed at 10% (by the total weight of cementitious ingredients). The flexural and compressive strengths of Series NS are shown in Figure 4a.

It can be observed that the flexural strength rose with the increase in nano-SiO_2_ content. When the nano-SiO_2_ content was 3.0%, the flexural strength reached 4.55 MPa, which was 1.98 times that of the reference group (NS-0). Aditionally, when the NS content was increased to 1.5%, the compressive strength increased, reaching 17.10 MPa, which was 2.25 times that of the reference group. As the NS content further increased to 3.0%, the compressive strength gradually declined. Figure 4b shows that the water absorption decreased with increasing nano-SiO_2_ content, while the softening coefficient exhibited a gradual increase. At a content level of 3.0%, the water absorption rate reached 26.97%, which was 0.76 times that of the reference group. Furthermore, the softening coefficient hit 0.47, which was 1.57 times that of the reference group.

The observed strength enhancement and water resistance improvement can be attributed to the incorporation of nano-SiO_2_ and Ca(OH)_2_ into calcined gypsum from phosphogypsum CGPCs. On the one hand, due to the high specific surface area of nano-SiO_2_, some nanoparticles reacted with calcium hydroxide (Ca(OH)_2_), which was added simultaneously, to form calcium silicate hydrates (C-S-H) within the gypsum composite paste. This reaction not only increased both the types and quantity of hydration products but also contributed positively to performance. On the other hand, unreacted nanosilica particles acted as micro-aggregates that filled voids among calcium sulfate dihydrate crystals, further reducing the porosity of the hardened paste. Consequently, this led to an improved microstructure of the hardened CGPCs, thereby enhancing their mechanical properties and water resistance [30,31]. However, an excessive NS content may result in the agglomeration and disruption of the crystal structure, ultimately hindering strength development [32].

#### 3.1.2. Effect of the Kaolin (KL) Content

**Figure 5:**
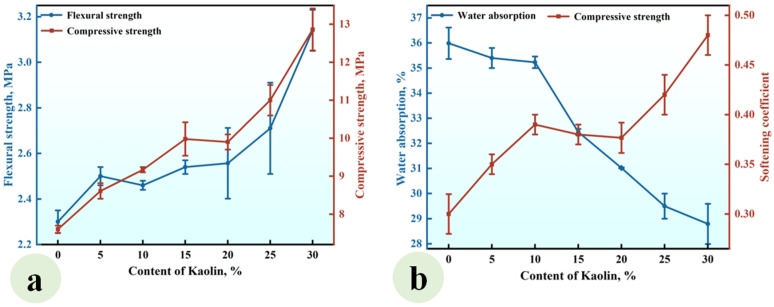
Effect of the KL content on (**a**) the strength and (**b**) water absorption and softening coefficient of the CGPCs.

A series of CGPCs, designated as Series KL, with mixed proportions specified in Table 2, were prepared to investigate the effect of the KL content on the properties of these materials. For each composite in Series KL, the content of CH was equivalent to that of kaolin. The results are presented in Figure 5.

As illustrated in Figure 5a, the flexural and compressive strengths exhibited an increase with the addition of kaolin, reaching values of 3.15 MPa and 12.60 MPa, respectively, when the kaolin content was 30%. These values were 1.37 and 1.66 times those of the reference group (KL-0). Figure 5b demonstrates that the water absorption of CGP decreased significantly with the increase in KL content, while the softening coefficient increased gradually. When the KL content increased from 0% to 30%, the water absorption decreased from 35.65% to 28.8%, and the softening coefficient increased from 0.30 to 0.48.

This phenomenon arose from the reaction between the added kaolin and Ca(OH)_2_, leading to the formation of insoluble products such as C-S-H. These products not only filled the voids among gypsum crystals, thereby enhancing the compactness of the hardened paste and improving its strength [33], but also adhered to or enveloped the surfaces of gypsum crystals, reducing their contact with water and subsequent dissolution, thus enhancing the water resistance of the hardened paste [34].

### 3.2. Effect of SP on the Properties of CGP

**Figure 6:**
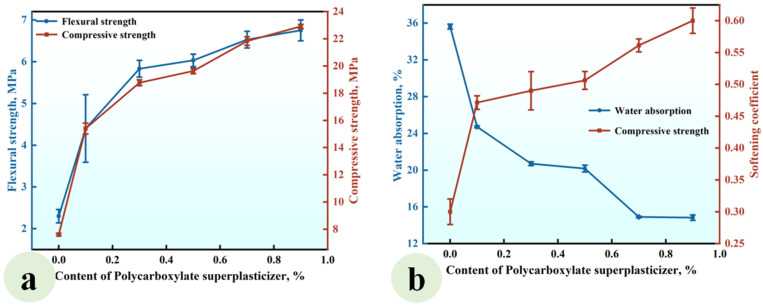
Effect of the SP content on (**a**) the strength and (**b**) water absorption and softening coefficient of CGP.

Figure 6 illustrates the experimental findings regarding the effect of the superplasticizer (SP) content (ranging from 0% to 0.9%) on the properties of CGP (Series SP), the formulations of which are listed in Table 2.

Figure 6a shows that the flexural and compressive strengths of CGP increased with increasing superplasticizer content. At a superplasticizer content of 0.9%, the flexural and compressive strengths reached values of 6.75 MPa and 22.95 MPa, respectively, reflecting increases of 193% and 202% compared to those observed in the reference group SP0 without superplasticizer. Figure 6b indicates that the water absorption of CGP progressively decreased with increasing superplasticizer content, while the softening coefficient exhibited a corresponding gradual increase. At a superplasticizer content of 0.9%, the water absorption attained a value of 14.83%, which was 58.4% lower than that recorded in the reference group (SP0). Conversely, the softening coefficient reached a value of 0.6, representing an increase of 100% compared to that observed in the reference group.

This observed phenomenon can be attributed to the dispersive action of the superplasticizer, which disrupted the flocculated structure of the CGP slurry. This disruption released water previously bound within the flocculated structure, resulting in a significant reduction in the water requirement for achieving standard consistency in CGP. Consequently, the porosity of the hardened CGP paste was diminished, leading to enhanced mechanical properties and improved water resistance [35,36].

### 3.3. Effect of Sodium Methyl Silicate (SMS) on the Properties of CGP

**Figure 7:**
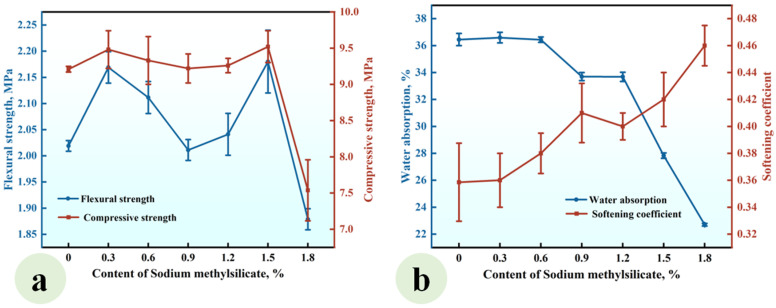
Effect of SMS content on (**a**) the strength and (**b**) water absorption and softening coefficient of CGP.

Series SMS was prepared to investigate the effect of the SMS content (ranging from 0% to 1.8%) on the properties of CGP. Its mixing proportions are listed in Table 2 and the experimental results are presented in Figure 7.

As depicted in Figure 7a, when the SMS content ranged from 0% to 1.5%, the flexural and compressive strengths of CGP exhibited fluctuations between 2.01 MPa and 2.18 MPa, as well as between 9.21 MPa and 9.52 MPa, respectively, indicating that within this content range, SMS exerted a limited effect on these strengths of CGP. However, an increase in the content from 1.5% to 1.8% led to a notable reduction in flexural strength from 2.18 MPa to 1.88 MPa, as well as a decrease in compressive strength from 9.52 MPa to 7.54 MPa, highlighting a significant deterioration in strength. In addition, Figure 7b demonstrates that as the SMS content increased, the water absorption of CGP progressively decreased. When the content rose from 0% to 1.2%, the reduction in water absorption was relatively gradual; however, with further increases in the content, this reduction became significantly more pronounced. At a content level of 1.8%, the water absorption dropped to the lowest value of 22.7%, which was markedly lower than that of SMS-0, the reference group (i.e., 36.5%). Concurrently, the softening coefficient exhibited a gradual increase with rising SMS content; specifically, as the content increased from 0% to 1.8%, the softening coefficient escalated from 0.36 to a maximum value of 0.46, indicating a substantial improvement in the water resistance of CGP. This can be attributed to the intricate physicochemical reactions that occurred in the CGP paste after the incorporation of sodium methyl silicate. The introduced sodium methyl silicate engaged in physicochemical reactions with water, resulting in the formation of a hydrophobic polysiloxane film on the surface of gypsum crystals and on the wall of pores within the hardened paste [35,36,37]. This film effectively inhibited the penetration of water into gypsum crystals, thereby significantly reducing the water absorption of the hardened paste and enhancing its water resistance. However, it simultaneously weakened the contact and bond between gypsum crystals, affecting both their morphology and microstructure [37,38,39,40,41]. Consequently, when the sodium methyl silicate content was high, a marked decrease in CGP strength was observed. Under the conditions established in this study, it is recommended that the SMS content be maintained at 1.5%.

### 3.4. Effect of Compounding of NS, SP, KL, and SMS on the Properties of the CGPCs

#### 3.4.1. Synergistic Effects of Integrating Nano-SiO_2_ with Superplasticizer

**Figure 8:**
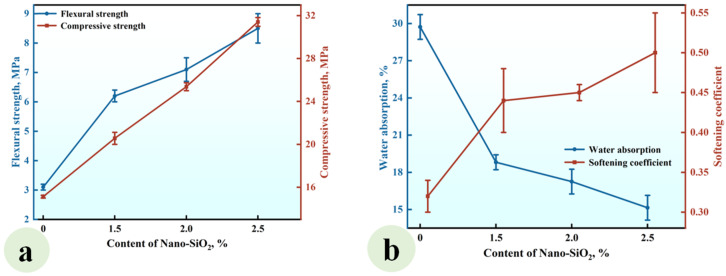
Effect of NS content on (**a**) the strength and (**b**) water absorption and softening coefficients of CGP incorporated with SP.

The experimental results detailed in Section 3.1.1 demonstrated that when the nano-SiO_2_ content was between 1.5% and 3.0%, with the simultaneous addition of CH, there was a marked improvement in both the strength and softening coefficient of CGP. Accordingly, this section explores the synergistic effects of integrating nano-SiO_2_ with polycarboxylate superplasticizer to further augment the water resistance and mechanical properties of CGP. The effect of different nano-SiO_2_ contents (specifically at 1.5%, 2.0%, and 2.5%) on the strength and water resistance of the CGPCs was examined, while maintaining a constant dosage of superplasticizer at 1.0%. The relevant findings are presented in Figure 8.

It can be seen from Figure 4 and Figure 8 that when nano-SiO_2_ was incorporated alongside the superplasticizer, the effect of the nano-SiO_2_ content on the properties of the CGPCs, including strength and water resistance, exhibited a similar trend to that observed when nano-SiO_2_ was added alone. As depicted in Figure 8a, both the flexural and compressive strengths of CGP increased progressively with increasing nano-SiO_2_ content. At a content of 2.5%, the flexural and compressive strengths reached values of 8.50 MPa and 31.41 MPa, respectively, representing increases of 174.2% and 107.6% compared to those of the reference group (SP-NS-0). Figure 8b demonstrates that the water absorption of the CGPCs decreased steadily as the nano-SiO_2_ content rose, while the softening coefficient exhibited an upward trend. Specifically, at a content level of 2.5%, the water absorption was merely 15.14%, reflecting a reduction of 49.06% from the reference group. Concurrently, the softening coefficient hit 0.50, representing an increase of 56.25% over that of the reference group. This can be attributed to two primary factors: first, the superplasticizer reduced the water requirement for standard consistency of CGP, thereby decreasing the porosity of the CGP hardened paste; second, due to both the pozzolanic effect and the microaggregate effect associated with nano-SiO_2_ (Section 3.5), the pores within the hardened paste were filled and further refined, resulting in the enhanced strength and water resistance of CGPC [34,41].

However, the above results show that although the addition of superplasticizer and nano-SiO_2_ significantly improved the strength and water resistance of CGP, its softening coefficient is still low (0.50). Therefore, further modification of CGP is needed to improve its water resistance.

#### 3.4.2. Synergistic Effects of Integrating Kaolin with Nano-SiO_2_ and Superplasticizer

**Figure 9:**
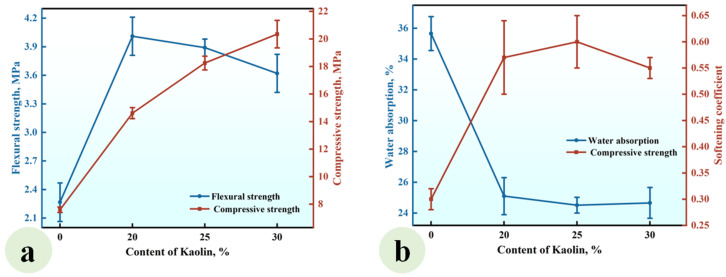
Effect of KL content on (**a**) the strength and (**b**) water absorption and softening coefficient of CGPCs incorporating SP and NS.

Series SP-NS-KL CGPC were prepared according to the formulations given in Table 2 to investigate the effects of varying kaolin contents (specifically at 20%, 25%, and 30% of the total weight of cementitious ingredients) on the water resistance and mechanical properties of the CGPCs. The experimental results are diagrammed in Figure 9.

Figure 9a shows that the flexural strength of Series SP-NS-KL initially increased and then decreased as the kaolin content increased. When the content was 20%, the flexural strength achieved a value of 4.00 MPa, reflecting an increase of 73.9% compared to that observed in the reference group (SP-NS-KL-0) without any additives. Additionally, the compressive strength increased with increasing KL content; at a kaolin content of 30%, it achieved a value of 20.35 MPa, representing an increase of 167.8% compared to that observed in the reference group. As indicated by Figure 9b, with the addition of KL, the water absorption of the CGPCs decreased while the softening coefficient exhibited an upward trend. At a KL content of 25%, the water absorption reached a value of 24.51%, which was 45.5% lower than that recorded in the reference group. Furthermore, the softening coefficient achieved a value of 0.6, representing an increase of 100% compared to that observed in the reference group.

This phenomenon can primarily be attributed to the pozzolanic effect related to kaolin, which was incorporated into the composites of Series SP-NS. Under the activation of calcium hydroxide present in the composites, kaolin and nano-SiO_2_ reacted with calcium hydroxide and calcined phosphorus gypsum, leading to the formation of hydration products such as C-S-H and ettringite (AFt) [33]. These products refined the pore structure of the hardened paste and enhanced its microstructure [35]. Consequently, the mechanical properties of the composites were improved, water absorption was reduced, and water resistance was enhanced. However, when the kaolin content exceeded a specific threshold, the quantity of AFt formed increased significantly. Given the micro-expansion characteristics of AFt crystals, an excessive quantity compromised the strength of the hardened paste, particularly its flexural strength. Therefore, it is essential to regulate the kaolin content within an optimal range (not exceeding 25% in this study).

#### 3.4.3. Synergistic Effects of Integrating Sodium Methyl Silicate with Nano-SiO_2_, Superplasticizer, and Kaolin

**Figure 10:**
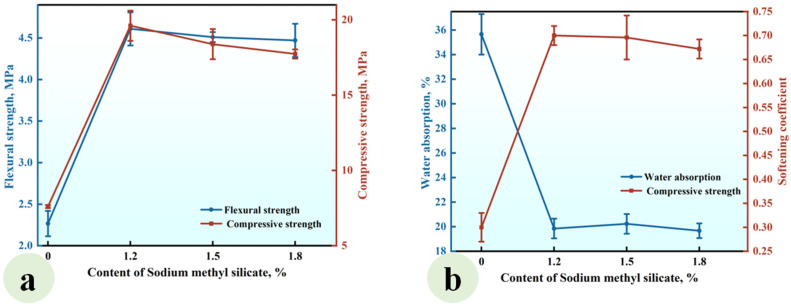
Effect of the SMS content on (**a**) the strength and (**b**) water absorption and softening coefficients of CGPCs incorporating SP, NS, and KL.

Sodium methyl silicate was introduced into Series SP-NS-KL to further improve its water resistance, and the effects of the sodium methyl silicate content on the strength, water absorption, and softening coefficient of the composites are examined in this section. The formulations of Series SP-NS-KL are given in Table 2. The SMS contents considered were 1.2%, 1.5%, and 1.8%, and the results are presented in Figure 10.

As illustrated in Figure 10a, the incorporation of SMS significantly enhanced both the flexural and compressive strengths of the CGPCs compared to the reference group. When the SMS content was 1.2%, the flexural and compressive strengths reached values of 4.61 MPa and 19.54 MPa, respectively, which were 2.00 and 2.57 times that of the reference group. This enhancement in strength can be attributed to the physical and chemical reactions induced by SMS within the CGP paste, which led to the formation of a hydrophobic film that adsorbed onto the surfaces of hydration products [15], particularly C-S-H. The film influenced the nucleation and growth of the gel product (C-S-H) to a certain extent, resulting in an increased number of gel particles and enhancing interparticle bonding, which positively contributed to the strength improvement of the hardened paste. However, when the SMS content was elevated to 1.5% and 1.8%, a slight decrease in strength was observed, which agreed with the results shown in Figure 7a. This decline can be ascribed to excessive SMS resulting in increased thickness of the film formed on the surface of the hydration product, which adversely affected gypsum crystal formation [15].

It can be observed in Figure 10b that the addition of methyl silicate sodium resulted in a significantly lower water absorption for the CGPCs compared to the reference group, while concurrently enhancing the softening coefficient. At an SMS content of 1.2%, the softening coefficient reached a value of 0.70, representing a 133.3% increase compared to that of the reference group. Under these conditions, the water absorption was measured at 19.85%, which was 44.32% lower than that recorded in the reference group. Furthermore, increasing the SMS content from 1.2% to 1.5% and subsequently to 1.8% did not yield significant changes in either the water absorption or softening coefficient. This can be attributed to the hydrophobic polysiloxane film formed on the surfaces of the hydration products and the walls of the pores within the hardened paste [35,36,37]. This film effectively inhibited water penetration into the hardened paste, thereby significantly reducing its water absorption and enhancing its water resistance. However, it simultaneously weakened the contact and bond between gypsum crystals, affecting both their morphology and microstructure [38,41].

Building upon the findings mentioned above, a highly water-resistant CGPC was successfully developed through the partial replacement of gypsum with kaolin and calcium hydroxide, the modification of nanosilica and sodium methyl silicate, and the incorporation of superplasticizer. The flexural and compressive strengths of the composite were measured at 4.61 MPa and 19.54 MPa, respectively, while the softening coefficient was determined to be 0.70, and the water absorption rate was recorded at 19.85%.

### 3.5. Microstructural Investigation and Water Contact Angle Test of Selected CGPCs

The microstructure of eight selected CGPCs, with formulations detailed in Table 3, was analyzed using the XRD and SEM-EDS techniques. Furthermore, the water contact angle test of the oven-dried specimens prepared according to the SP-NS-KL-SMS-1.2 formula was performed utilizing an automatic contact angle tester.

#### 3.5.1. XRD

**Figure 11:**
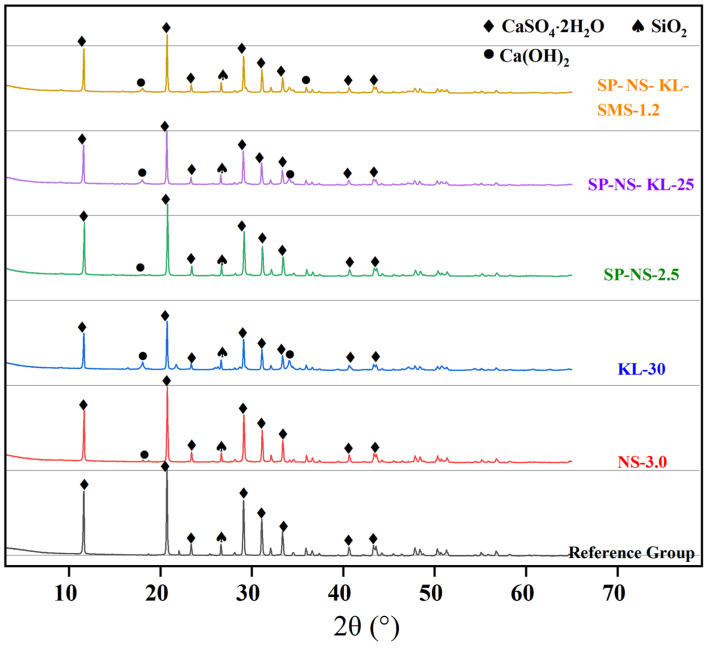
XRD patterns of the hydration samples of the selected CGP composites.

Figure 11 shows the XRD patterns of the hydration samples of the selected CGP, i.e., composites NS-3.0, KL-30, SP-NS-2.5, SP-NS-KL25, SP-NS-KL-SMS-1.2, and the reference group.

Diffraction peaks corresponding to calcium sulfate dihydrate crystals appeared in all hydration samples of the selected CGPCs, confirming that incorporating admixtures (mineral or chemical) did not alter the main hydration product of calcined phosphogypsum. Additionally, the intensity of the peaks (11.6°, 20.7°, and 29.1°) belonging to calcium sulfate dihydrate crystals in composites KL-30, SP-NS-KL-25, and SP-NS-KL-SMS-1.2 was noticeably lower than that in the reference group. This implied that fewer gypsum crystals were formed in the hydration samples of these composites, a result of the partial substitution of CGP with kaolin and nano-SiO_2_. However, no characteristic diffraction peaks of kaolinite, the primary component of kaolin, were detected in these hydration samples, likely due to the consumption of kaolinite during its pozzolanic reaction with calcium hydroxide. Furthermore, no calcium silicate hydrates (C-S-H), the products of pozzolanic reaction, were identified in these samples, which can be attributed to the low crystallinity of C-S-H, classified as a kind of gel product. Nevertheless, the presence of C-S-H was revealed by the results of SEM (see Section 3.5.2).

#### 3.5.2. SEM

**Figure 12:**
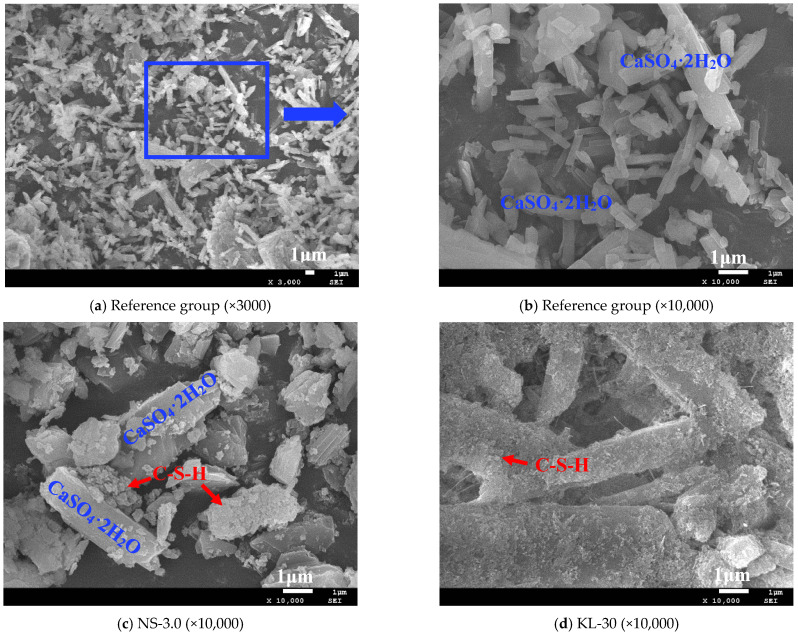

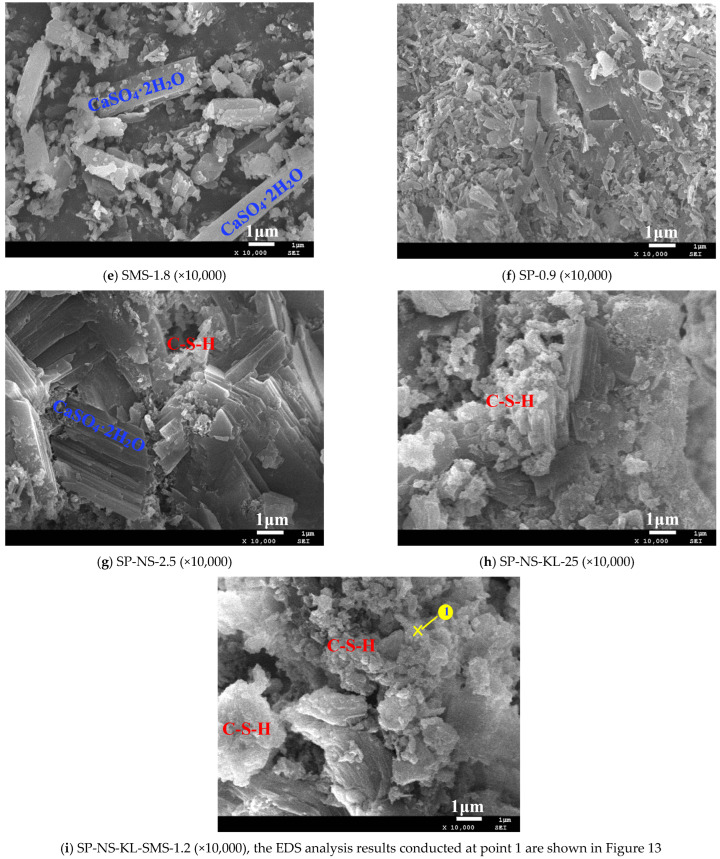
SEM images of the CGPCs incorporating different additives.

The morphology of the hydration products and microstructures of the selected CGPCs incorporated with different additives were investigated using SEM; the SEM images are shown in Figure 12.

As depicted in Figure 12, calcium sulfate dihydrate crystals were the main hydration product of these CGPCs, which agrees with the XRD results (Figure 11). However, the incorporation of additives into the composites resulted in notable changes in both the types and morphology of their hydration products. Figure 12a,b demonstrate that in the control group, gypsum crystals predominantly exhibited columnar or plate-like morphologies; however, there was significant variability in crystal size, accompanied by substantial pores between the crystals, which likely adversely affected the strength of the hardened paste. This phenomenon elucidates why the strength of the control group is comparatively lower from a microstructural perspective. Upon the addition of nano-SiO_2_ (see Figure 12c) or KL (see Figure 12d), a small quantity of gel products was observed to adsorb onto the surfaces of gypsum crystals within the hydrated samples. Notably, when KL was incorporated, there was a substantial increase in the number of gel products adhering to the surfaces of gypsum crystals. This can be attributed to the pozzolanic reaction between nano-SiO_2_ or KL and the concurrently added calcium hydroxide (CH), resulting in C-S-H formation [42,43,44]. Furthermore, since the amount of KL introduced significantly exceeded that of nano-SiO_2_ (refer to Table 2 and Table 3), this led to a pronounced increase in the C-S-H quantities formed.; when an appropriate dosage of superplasticizer was applied alone (see Figure 12f), no significant alterations were noted in either the shape or size of gypsum crystals. However, the voids between the crystals markedly diminished while the compactness of the paste improved substantially, which can be attributed to the fact that incorporating the SP reduced the water demand for standard consistency of the slurry, thereby contributing to notable enhancements in the strength and water resistance of the hardened paste [33,44] (as detailed in Section 3.2 and Figure 6). When two (see Figure 12g) or more types (see Figure 12h,i) of additives were simultaneously introduced, all aforementioned microstructural improvements manifested. On the one hand, new hydration products such as C-S-H emerged within the paste; on the other hand, gypsum crystals alongside C-S-H became more densely arranged, resulting in increased compactness, which inevitably enhanced both the strength and water resistance of the CGPCs.

**Figure 13:**
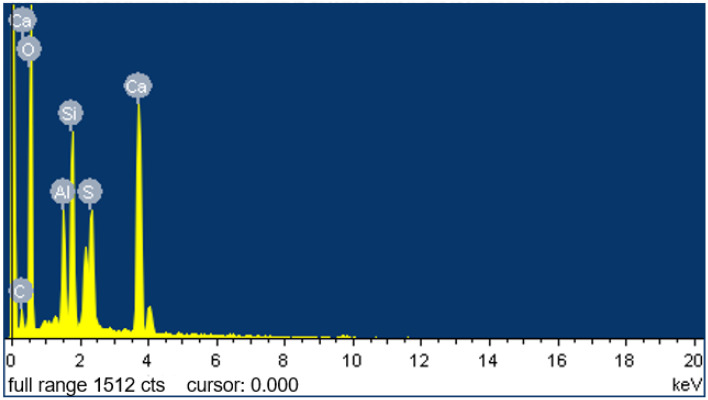
Results of the EDS analysis conducted at Spot 1, which was provided in Figure 12i.

To explore the changes in the microstructure of the CGP paste following the addition of SMS, Spot 1, provided in Figure 12i, was selected for EDS analysis. The results are presented in Figure 13 and Table 4.

The results confirmed the existence of calcium silicate hydrate (C-S-H) within the hydration samples, and the identification of carbon (C) corroborated the presence of functional groups containing carbon on the surface of the hydration products, such as C-S-H and gypsum crystals. Therefore, based on the results of the EDS analysis, it can be inferred that following the incorporation of SMS into the CGPCs, a polysiloxane film was formed on the surface and pore walls of the hardened matrix [15]. This hydrophobic film effectively inhibited water penetration into the hardened matrix [41,42], thereby partially elucidating the observed reduction in the water absorption rate and the increase in the softening coefficient after SMS addition (refer to Section 3.3 and Section 3.4.3).

#### 3.5.3. Water Contact Angle

**Figure 14:**
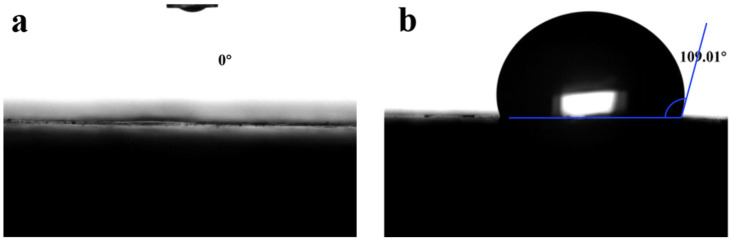
Water contact angle testing for specimens of (**a**) the reference group and (**b**) the SP-NS-KL-SMS-1.2 composite.

The outcomes of the water contact angle assessment for the oven-dried specimens from both the reference group and the SP-NS-KL-SMS-1.2 composite are illustrated in Figure 14.

It can be observed from Figure 14a that the surface of the reference group specimen exhibited a water contact angle of 0°, indicating exceptionally strong hydrophilicity and facilitating effective water infiltration [44]. In contrast, the SP-NS-KL-SMS-1.2 specimen displayed a contact angle of 109.01° (see Figure 14b), signifying that the CGPC modified by additives demonstrated significant hydrophobicity. This phenomenon is primarily attributed to SMS incorporation, which promotes the formation of a hydrophobic polysiloxane film on the surface of the hydration products within the CGPC, thereby effectively preventing further penetration of water into its interior, thereby significantly enhancing its water resistance.

### 3.6. Performance Evaluation and Cost Analysis of the CGPCs

#### 3.6.1. Performance Evaluation and Challenges of Large-Scale Application of CGP Composites

Based on the aforementioned findings, Table 5 presents a comprehensive summary of the water resistance, mechanical properties, and optimal formulation of CGPCs, and compares these properties with those of high-strength gypsum powder.

As shown in Table 5, the compressive strength of the optimally formulated CGPCs reached 19.5 MPa, with a softening coefficient of 0.70. In contrast, conventional CGP exhibited a compressive strength of only 7.60 MPa and a softening coefficient of 0.30. Although high-strength gypsum powder achieved a compressive strength of 20–30 MPa, its softening coefficient remained relatively low at 0.35–0.45. Taking both water resistance and strength into account, the CGPCs demonstrated significant advantages in terms of water resistance.

It is important to note that the performance data of the CGPCs presented in Table 5 are based on laboratory findings. To facilitate the large-scale practical application of CGPCs, it is necessary to address the following challenges and conduct systematic, in-depth scientific investigations: (1) enhance the measurement accuracy of each constituent; (2) ensure the homogeneity of the mixture of all components; (3) perform a systematic evaluation of long-term performance and durability; (4) carry out a comprehensive analysis of long-term environmental impacts; (5) optimize cost control strategies.

#### 3.6.2. Cost Analysis of the CGPCs

Based on the market prices of each component [46], a cost analysis was performed for the optimally formulated CGPCs (SP-NS-KL-SMS-1.2), based on the results presented in detail in Table 5.

As indicated in Table 6, the total cost of the CGPCs was approximately CNY 625 per ton, which is significantly higher than that of calcined gypsum from phosphogypsum (CNY 380 per ton) but lower than that of high-strength gypsum powder (CNY 820 per ton). Taking both cost and properties into account (Table 5 and Table 6), the CGPCs demonstrate significant advantages in practical applications.

## 4. Conclusions

The present study sought to enhance the water resistance and mechanical strength of CGP by incorporating inorganic mineral admixtures (kaolin, nanosilica, and hydrated lime) or organic additives (polycarboxylic acid superplasticizer and sodium methyl silicate), thereby facilitating the recycling and comprehensive utilization of phosphogypsum. The effects of the dosage of these additives, including nanosilica, kaolin, polycarboxylic acid superplasticizer, and sodium methyl silicate, on the properties (flexural strength, compressive strength, water absorption, and softening coefficient) of CGPCs were studied. Their influence mechanisms were also investigated using the XRD and SEM-EDS analyses and water contact angle assessment. The following conclusions were drawn from this study:

(a) The incorporation of nanosilica into CGP significantly improved the material’s mechanical strength and water resistance. The flexural strength, compressive strength, and softening coefficient of the CGPCs exhibited a significant increase with increasing nanosilica content. When the nanosilica content was 3%, the flexural and compressive strengths of the CGPCs were 4.55 MPa and 16.92 MPa, respectively. However, its softening coefficient was still relatively low, at only 0.47.
(b) Incorporating kaolin also resulted in the enhancement of the mechanical strength and water resistance of the CGPCs. When the kaolin content was 30%, the flexural and compressive strengths exhibited an increase with kaolin content, reaching 3.15 MPa and 12.60 MPa, respectively. Nevertheless, the softening coefficient was only 0.48.
(c) The addition of superplasticizer demonstrated substantial advantages in enhancing the strength and water resistance of CGP. At a superplasticizer content of 0.9%, the flexural and compressive strengths of CGP reached 6.75 MPa and 22.95 MPa, respectively. Additionally, the softening coefficient was measured at 0.60.
(d) Modification with sodium methyl silicate significantly improved the water resistance of CGP, while having a limited effect on mechanical strength when its content varied from 0% to 1.5%. At a sodium methyl silicate content of 1.5%, the softening coefficient reached 0.42.
(e) Microstructural investigation confirmed that the incorporation of nanosilica, kaolin, and hydrated lime into CGP enhanced the microstructure of the CGP paste. Additionally, the superplasticizer played a crucial role in reducing the water-to-binder ratio, thereby enhancing the microstructure of the hardened paste of the CGPCs. Additionally, the sodium methyl silicate formed a hydrophobic film on the surface of the hardened paste, increasing the contact angle to 109.01° and improving the water resistance of CGP. Consequently, both the mechanical strength and water resistance of the CGPCs exhibited marked improvement.
(f) The high water resistance of the CGPCs was achieved through modification with nanosilica and sodium methyl silicate, the addition of superplasticizer, and the partial replacement of gypsum with kaolin and hydrated lime, and its optimal proportion, i.e., the mass ratio of calcined phosphogypsum, kaolin, hydrated lime, nanosilica, superplasticizer, and sodium methyl silicate, was 75:25:25:2.5:1:1.2. The results showed that its flexural and compressive strengths reached 4.61 MPa and 19.54 MPa, respectively, while its softening coefficient was 0.70.

## Figures and Tables

**Table 1:** Chemical composition of CGP and KL (wt/%).

Chemical Composition	SO_3_	CaO	SiO_2_	Al_2_O_3_	Fe_2_O_3_	P_2_O_5_	F	TiO_2_	LOI
CGP	43.40	36.11	8.98	0.57	0.68	0.59	0.68	-	8.47
KL	-	-	53.03	41.30	0.81	-	-	1.00	-

**Table 2:** Mix proportions of the CGPCs.

Composite Number	Constituents of CGP (wt/%)			Additive (wt/%)		
	CGP	KL	CH	NS	SP	SMS
KL-0	100	0	0	-	-	-
KL-5	95	5	5	-	-	-
KL-10	90	10	10	-	-	-
KL-15	85	15	15	-	-	-
KL-20	80	20	20	-	-	-
KL-25	75	25	25	-	-	-
KL-30	70	30	30	-	-	-
NS-0	100	-	0	0.0	-	-
NS-0.5	100	-	10	0.5	-	-
NS-1.0	100	-	10	1.0	-	-
NS-1.5	100	-	10	1.5	-	-
NS-2.0	100	-	10	2.0	-	-
NS-2.5	100	-	10	2.5	-	-
NS-3.0	100	-	10	3.0	-	-
SP-0	100	-	-	-	0	-
SP-0.1	100	-	-	-	0.1	-
SP-0.3	100	-	-	-	0.3	-
SP-0.5	100	-	-	-	0.5	-
SP-0.7	100	-	-	-	0.7	-
SP-0.9	100	-	-	-	0.9	-
SMS-0	100	-	-	-	-	0
SMS-0.3	100	-	-	-	-	0.3
SMS-0.6	100	-	-	-	-	0.6
SMS-0.9	100	-	-	-	-	0.9
SMS-1.2	100	-	-	-	-	1.2
SMS-1.5	100	-	-	-	-	1.5
SMS-1.8	100	-	-	-	-	1.8
SP-NS-0	100	-	10	0	1.0	-
SP-NS-1.5	100	-	10	1.5	1.0	-
SP-NS-2.0	100	-	10	2.0	1.0	-
SP-NS-2.5	100	-	10	2.5	1.0	-
SP-NS-KL-0	100	0	0	0	0	-
SP-NS-KL-20	80	20	20	2.5	1.0	-
SP-NS-KL-25	75	25	25	2.5	1.0	-
SP-NS-KL-30	70	30	30	2.5	1.0	-
SP-NS-KL-SMS-0	100	0	0	0	0	0
SP-NS-KL-SMS-1.2	75	25	25	2.5	1.0	1.2
SP-NS-KL-SMS-1.5	75	25	25	2.5	1.0	1.5
SP-NS-KL-SMS-1.8	75	25	25	2.5	1.0	1.8

**Table 3:** Formulations of the selected CGPCs for microstructural investigation and water contact angle testing.

Composite No.	Ingredients (wt/%)			Additive (wt/%)			Items
	CGP	KL	CH	NS	SP	SMS	
Reference group *	100	-	-	-	-	-	XRD, SEM
NS-3.0	100	-	10	3.0	-	-	XRD, SEM
KL-30	70	30	30	-	-	-	XRD, SEM
SP-0.9	100	-	-	-	0.9	-	SEM
SMS-1.8	100	-	-	-	-	1.8	SEM
SP-NS-2.5	100	-	10	2.5	1.0	-	XRD, SEM
SP-NS-KL-25	75	25	25	2.5	1.0	-	XRD, SEM
SP-NS-KL-SMS-1.2	75	25	25	2.5	1.0	1.2	XRD, SEM-EDS, water contact angle

* As implied in Table 2, the reference groups can also be designated as NS-0, KL-0, SP-0, SMS-0, SP-NS-KL-0, and SP-NS-KL-SMS-0.

**Table 4:** Elemental content detected at Spot 1, which was provided in Figure 12i, via EDS.

Element	Weight Percentage, %	Atomic Percentage, %
C	0.16	0.27
O	58.37	74.67
Al	5.17	3.92
Si	9.23	6.73
S	4.61	2.94
Ca	22.46	11.47

**Table 5:** Properties of conventional gypsum-based materials (including calcined gypsum derived from phosphogypsum and high-strength gypsum) and the CGPCs developed in this study.

Composite No.		Flexural Strength, MPa	Compressive Strength, MPa	Water Absorption, %	Softening Coefficient
Conventional gypsum-based materials	Calcined gypsum from phosphogypsum	2.30	7.60	35.65	0.30
	High-strength gypsum [23,27,45]	3.5–4.5	20–30	/	0.30–0.45
Optimally formulated CGPCs (SP-NS-KL-SMS-1.2)		4.61	19.54	19.85	0.70

**Table 6:** Cost analysis of the optimally formulated CGP composites developed in this study.

	Components	CGP	KL	CH	NS	SP	SMS
Item							
Mass percentage, %		75	25	25	2.5	1.0	1.2
Component price [46], CNY/ton		380	400	200	4900	1800	4000
Component cost, CNY		285	100	50	122.5	18	48
Total cost, CNY/ton		623.5					

## Data Availability

The original contributions presented in the study are included in the article, and further inquiries can be directed to the corresponding author.

## References

[1] Yang J., Ma L., Liu H., Guo Z., Bounkhong K. (2020). Chemical behavior of fluorine and phosphorus in chemical looping gasification using phosphogypsum as an oxygen carrier. Chemosphere.

[2] Zhou J., Li X., Zhao Y., Shu Z., Shen X. (2020). Preparation of paper-free and fiber-free plasterboard with high strength using phosphogypsum. Constr. Build. Mater..

[3] Li B., Shu J., Chen M., Zeng X., Liu R., Yang Y. (2023). A new basic burning raw material for simultaneous stabilization/solidification of PO_4_^3−^-P and F^−^ in phosphogypsum. Ecotox Environ. Safe.

[4] Singh M. (2002). Treating waste phosphogypsum for cement and plaster manufacture. Cem. Concr. Res..

[5] Chen X., Gao J., Zhao Y. (2019). Investigation on the hydration of hemihydrate phosphogypsum after post treatment. Constr. Build. Mater..

[6] Romero-Hermida M.I., Flores-Alés V., Hurtado-Bermúdez S.J., Santos A., Esquivias L. (2020). Environmental impact of phosphogypsum-derived building materials. Int. J. Environ. Res. Public Health.

[7] Wei Z., Deng Z. (2022). Research hotspots and trends of comprehensive utilization of phosphogypsum: Bibliometric analysis. J. Environ. Radioact..

[8] Tayibi H., Choura M., Lopez F.A., Alguacil F.J., Lopez-Delgado A. (2009). Environmental impact and management of phosphogypsum. J. Environ. Manage..

[9] Rashad A.M. (2017). Phosphogypsum as a construction material. J. Clean. Prod..

[10] Chernysh Y., Yakhnenko O., Chubur V., Roubík H. (2021). Phosphogypsum recycling: A review of environmental issues, current trends, and prospects. Appl. Sci..

[11] Wu F., Liu X., Wang C., Qu G., Liu L., Chen B. (2022). New dawn of solid waste resource treatment: Preparation of high-performance building materials from waste-gypsum by mechanical technology. Constr. Build. Mater..

[12] Tzouvalas G., Rantis G., Tsimas S. (2004). Alternative calcium-sulfate-bearing materials as cement retarders: Part II. FGD gypsum. Cem. Concr. Res..

[13] Wu Q., Ma H., Chen Q., Huang Z., Zhang C., Yang T. (2019). Preparation of waterproof block by silicate clinker modified FGD gypsum. Constr. Build. Mater..

[14] Fornes I.V., Vaičiukynienė D., Nizevičienė D., Doroševas V., Dvořák K. (2021). A method to prepare a high-strength building material from press-formed phosphogypsum purified with waste zeolite. J. Build. Eng..

[15] He T., Kang Z., Chen C. (2021). Influence of Sodium Methyl Silicate on Waterproof Property of Desulfurized Gypsum Block. J. Build. Mater..

[16] Kaziliunas A., Leskeviciene V., Vektaris B., Valancius Z. (2006). The study of neutralization of the dihydrate phosphogypsum impurities. Ceram. Silik..

[17] Sui S., Li J., Guan R., Wang D., Li G. (2005). Research on Water Resistance Performance of Gypsum Products. J. Build. Mater..

[18] Pan H., Li G. (2013). Emulsion Waterproof Agent and Its Effects on Intrinsic Properties of Gypsum. Asian J. Chem..

[19] Lin Z., Xing W., Chen W. (2014). Cementitious Materials Science.

[20] Chen Y., Yue W., Dong R. (2003). Gypsum Building Materials.

[21] Yuan R. (1989). Cementitious Materials.

[22] Cao J., Li J., Jiang Y., Wang S., Ding Y., Ding Y., Oh W. (2019). Improvement in water resistance of desulfurized gypsum by novel modification of silicone oil paraffin composite emulsion-based waterproofing agent. J. Korean Ceram. Soc..

[23] Wang W. (2023). Study on Improvement Method of Water-Resistance of Hemihydrate Phosphogypsum.

[24] Liu D., Wang W., Peng Y., Shi H., Li D., Wang B. (2022). Study on the Strength and Hydration Characteristics of Phosphogypsum-phosphorus Slag Composite Cementitious Material. Met. Mine.

[25] Li J. (2020). Influence of Silica Fume on Properties of Gypsum-based Self-leveling Mortar. China Concr. Cem. Prod..

[26] Liu K., Wang A., Sun D., Chen W. (2016). Recent Progress of Ettringite Formation and Its Expansion Mechanisms during Sulfate Attack. Bull. Chin. Ceram. Soc..

[27] Pan Z., Chen Y., Wu J., Tan Y. (2023). Study on the Behavior of Swelling-Shrinkage in Hydration Process of Phosphogypsum-based super Sulfated Cement. J. China Three Gorges Univ. Nat. Sci..

[28] Zhang W., Yuan Q., Liu X., Mou X. (2018). Application of Silane Materials in Concrete Protection. Constr. Sci. Technol..

[29] (1999). Gypsum Plasters-Determination of Mechanical Properties.

[30] Li J., Cao J., Ren Q., Ding Y., Zhu H., Xiong C., Chen R. (2021). Effect of nano-silica and silicone oil paraffin emulsion composite waterproofing agent on the water resistance of flue gas desulfurization gypsum. Constr. Build. Mater..

[31] Zhang Y., Tao Z., Wu L., Zhang Z., Zhao Z. (2023). Study on Effect of Nano-CaCO_3_ on Properties of Phosphorus Building Gypsum. Materials.

[32] Tokarev Y., Ginchitsky E., Sychugov S., Krutikov V., Yakovlev G., Buryanov A., Senkov S. (2017). Modification of gypsum binders by using carbon nanotubes and mineral additives. Procedia Eng..

[33] Fraire-Luna P.E., Escalante-Garcia J.I., Gorokhovsky A. (2006). Composite systems fluorgypsum-blastfurnance slag-metakaolin, strength and microstructures. Cem. Concr. Res..

[34] Guan B., Ye Q., Zhang J., Lou W., Wu Z. (2010). Interaction between α-calcium sulfate hemihydrate and superplasticizer from the point of adsorption characteristics, hydration and hardening process. Cem. Concr. Res..

[35] Li Z., Xu K., Peng J., Wang J., Zhang J., Li Q. (2021). Study on mechanical strength and water resistance of organosilicon waterproofing agent blended recycled gypsum plaster. Case Stud. Constr. Mat..

[36] Wu Q., Ma H., Chen Q., Gu B., Li S., Zhu H. (2019). Effect of silane modified styrene-acrylic emulsion on the waterproof properties of flue gas desulfurization gypsum. Constr. Build. Mater..

[37] Chen C., Ma F., He T., Kang Z., Wang Y., Shi C. (2022). Improved water and efflorescence resistance of flue gas desulfurization gypsum-based composites by generating hydrophobic coatings. J. Clean. Prod..

[38] Cui G., Kong D., Huang Y., Qiu W., Cheng L., Wang L. (2023). Effects of Different Admixtures on the Mechanical and Thermal Insulation Properties of Desulfurization Gypsum-Based Composites. Coatings.

[39] Ekaterina F., Elena V., Oleg B., Andreev V. (2016). Some aspects on improvement of water resistant perfornace of gypsum binders. Matec Web Conf..

[40] Kumagai S., Ohama Y. (2002). Development of Highly Water-Resistant Gypsum-Based Composites. Zairyo J. Soc. Mater. Sci..

[41] Li L., Li B., Chen P., Yin S. (2022). Modification and Mechanism of Phosphorus Building Gypsum Using Admixtures. Bull. Chin. Ceram. Soc..

[42] Zhang S., Yang B., Zhai W., Li S., Kong G. (2021). Composition and properties of methyl silicate/silicate composite coatings. J. Mater. Eng..

[43] Min J., Park J.H., Sohn H.K., Park J.M. (2012). Synergistic effect of potassium metal siliconate on silicate conversion coating for corrosion protection of galvanized steel. J. Ind. Eng. Chem..

[44] Giovambattista N., Debenedetti P.G., Rossky P.J. (2007). Effect of surface polarity on water contact angle and interfacial hydration structure. J. Phys. Chem. B.

[45] Tian W., Wang Q., Zhang Y., Xu F., Chen S. (2020). Study on the Properties of Phosphogypsum Mixed with Sulphoaluminate Cement for Concrete Canvas. J. China Three Gorges Univ. Nat. Sci..

[46] http://zj.yichang.gov.cn/content-62527-985084-1.html.

